# Melasma and its association with different types of nevi in women: A case-control study

**DOI:** 10.1186/1471-5945-8-3

**Published:** 2008-08-05

**Authors:** Hassan Adalatkhah, Homayoun Sadeghi-bazargani, Nayereh Amini-sani, Somayeh Zeynizadeh

**Affiliations:** 1 Dermatology department, Imam Khomeini University hospital,Ardabil University of medical sciences, Ardabil, Iran; 2 Department of public health sciences, Karolinska Institute, Stockholm, Sweden; 3 NPMC & Research development center (RDCC), Tabriz University of medical sciences, Tabriz, Iran

## Abstract

**Background:**

Very little is known about possible association of nevi and melasma. The study objective was to determine if there is an association between melasma and existence of different kinds of nevi.

**Methods:**

In a case-control study, 120 female melasma patients referred to dermatology clinic of Ardabil and 120 patients referred to other specialty clinics who lacked melasma were enrolled after matching for age. Number of different types of nevi including lentigines and melanocytic nevi were compared between case and control group patients. Data were entered into the computer and analyzed by SPSS 13 statistical software.

**Results:**

Mean number of lentigines was 25.5 in melasma group compared to 8 in control group(P < 0.01). Mean number of melanocytic nevi was 13.2 in cases compared to 2.8 in control group(P < 0.001). Multivariate analysis showed that existence of freckles, lentigines and more than three melanocytic nevi were positively related to developing melasma. The chance of melasma increased up to 23 times for patients having more than three melanocytic nevi. Congenital nevi were observed among 10% both in case and control groups. Campbell de morgan angiomas were seen among 26 patients(21.8%) in case group compared to 6 patients(5%) in control group.

**Conclusion:**

Existence of lentigines and melanocytic nevi increases chance of having melasma

## Background

Facial appearance plays a large role in self-perception and interaction with others and severe facial blemishes like melasma leave a significant impact on women's quality of life [1]. Melasma is an acquired hypermelanosis, occurring symmetrically on sun-exposed areas of the body. Lesions are irregular light to dark brown macules and patches, usually involving the forehead, temples, upper lip, and cheeks [2]. Asian and Hispanic females are most commonly affected [2,3]. Several factors have been found to be in relation with melasma. Nevi are usually long lasting and have an earlier presentation than melasma. Very little research is done to study possible associations between different types of nevi and melasma and to the best of our knowledge no other study has got focused on prediction value of different kinds of nevi for future melasma. Our aim of study was to find out if there is an association between the existence of different kinds of nevi and melasma.

## Methods

In a case-control study, 120 female patients referred to dermatology clinic of Ardabil due to melasma and 120 patients referred to other specialty clinics who lacked melasma were enrolled. All of the melsama group patients who were asked to participate in this study accepted to participate. Only three people in control group didn't participate and were replaced by others. Using frequency matching technique, controls were matched for age. Sample size was calculated based on data coming from our pilot study to fulfill the least power of 0.8 and a significane level 0.05 for predefined bivariate proportion comparison test, regarding exposure proportions of melanocytic nevi and lentigens using the formula presented by lemshaw and correcting for multiple test effect on type one error.

The questionnaire included three parts to collect data regarding demographic information, medical history and medical examination. Both the interview and medical examination was done by a dermatologist. We didn't include any questions regarding sun exposure, just due to similar clothing and cover used by Iranian women. A melasma diagnosis was made based on clinical examination. Different types of nevi were also diagnosed clinically [4]. As it is often impossible to distinguish lentigines from junctional or flat compound nevi on clinical grounds, so to decrease the limitations of clinical diagnosis in differentiating some cases of lentigines from melanocytic nevi, a combination analysis was also done [4].

Data were entered into the computer and analyzed by SPSS 13 statistical software. Means were compared using t test and proportions were compared using chi-square test. Crude and adjusted odds ratios were calculated along with 95% confidence intervals. A logistic regression model was constructed after checking for necessary model diagnostics. Age was matched between groups. Demographic variables lacking statistically significant distribution difference were not entered into the model.

The study was approved by committee of ethics in Ardabil University of medical sciences. After declaring sufficient information, a verbal consent was taken from all participants. Nearly total body inspection was made by a female physician to meet cultural limitations.

## Results

Demographic information compared between case and control groups are given in Table 1. Centro-facial pattern was observed among 95 percent of melasma patients and five percent of them had malar pattern melasma. None of the patients had a mandibular pattern melasma. 96.7 percent of participants in our study were clinically categorized to have type two and type three phototypes. Type three phototype constituted more than two-third of all participants without significant difference between case and control groups. 189 of the total participants were urban residents, mostly housewives or employees. They lived in religious city of Ardabil and they wore similar Islamic clothes leaving only the hands and partially the face to be uncovered.

**Table 1:** Some demographic and medical history information compared between case and control groups

Factors/Groups & statistics		Cases		Controls		Statistical significance
				
		Frequency/Mean	Percent/Standard deviation	Frequency/Mean	Percent/Standard deviation	
Age		29.97	6.6	29.68	6.7	NS*

Marital status	Married	93	77	85	71	NS
	Single	27	23	35	29	

Education	Academic	13	11.3	23	19.3	NS
	Non academic	117	88.7	97	80.7	

Known melasma related diseases		0	0	0	0	7

• *: Non significant at 0.05 level

• **: Hypovitaminosis, Ovarian diseases, Specific endocrinologic diseases

24 of the melasma patients and 27 of the control group participants were rural residents being more exposed to sun in summer and harvest time compared to urban participants but without statistical difference between study groups.

### Freckels

29 patients (24.3%) in case group compared to five (4.16%) in control group had freckles (P < 0.001).

### Lentigines

77 patients (64.1%) in case group had lentigines compared to 20(16.6%) in control group(P < 0.001). Mean number of lentigines was 25.5 compared to 8 in control group(P < 0.01). The common lentigo involvment pattern was head and neck involvement in case group and involvement of the hands in control group.

### Melanocytic nevi

Melanocytic nevi were observed among more than 95% of patients in both groups. But mean number of melanocytic nevi was 13.2 in cases compared to 2.8 in control group(P < 0.001).

### Congenital nevi

Congenital nevi were observed among 10% in both case and control groups.

### Campbell de morgan angiomas

These were seen among 26 patients(21.8%) in case group compared to 6 patients(5%) in control group(P < 0/001). Mean number of these angiomas was 1 in control group and 5.2 in case group (P = 0.02). The common Campbell de morgan angioma involvement pattern was trunk involvement (57%) in case group and head and neck involvement(50%) in control group.

### Other nevi

Other types of nevi observed were skin tags, Café-au-lait spots and nevus anemicus without any significant distribution between groups. Neither epidermal nor dysplastic nevi were observed in our study participants.

### Independent associations of nevi and melasma

Multivariate regression analysis showed that having freckles, lentigines and having more than three melanocytic nevi were positively associated with melasma in women. The chance of melasma increases up to 23 times for people having more than three melanocytic nevi. Crude and adjusted odds ratios of the occurrence of melasma for patients having different types of nevi are given in Table 2.

**Table 2:** Melasma association with different types of nevi

**Exposure**	**Exposure percentage**		**Crude odds ratios**			**Adjusted odds ratios**		
			
	**Percent in cases**	**Percent in controls**	**OR**	**95% CI of OR**	**Significance**	**OR**	**95% CI of OR**	**Significance**
**Freckle**	24.3	4.16	7.41	2.6 – 22.8	S	5.9	1.5 – 23.7	S
**Lentigines**	64.1	16.6	9.17	4.8 – 17.7	S	5.2	1.7 – 9.3	S
**Above 3 Melanocytic nevi**	90.7	22.3	33.67	14.9 – 77.8	S	23	9.7 – 54.7	S
**Campbell de morgan angiomas**	21.8	5	5.3	2–15.08	S	3.2	0.8 – 13.5	NS
**Other nevus types**	4.1	6.6	.6	0.2 – 2.1	NS	0.9	0.2 – 4.9	NS
**Melasma in close relatives**	64.1	38.3	2.09	1.6 – 5.04	S	1.6	0.7 – 3.7	NS

S: Statistically significant. NS: Statistically non-significant

To check the effect of misdiagnosis between lentigines and some of melanocytic nevi, we entered a new variable into the analysis model representing the existence of either lentigines or more than three melanocytic nevi. This resulted in even higher odds ratio compared to having freckles.

## Discussion

In our study, 64 percent of melasma patients had a positive family history. Positive family history of melasma is reported in several studies [5-9]. Although, genetic influence is suggested by a twin study but it is not generally accepted [10,11].

Melanocytic nevi existed among 95% of patients in both groups of our study. Studying 432 healthy Caucasian subjects, MacKie found the mean body nevus count during first decade of life to be three for females and two for males, rising rapidly up to a mean of 33 for females and 22 for males in the third decade. Thereafter, numbers of moles slowly dropped until in the eighth decade; the count fell to levels similar to those seen in pre-pubertal children [12]. In an Australian study, the mean number of melanocytic nevi was as high as 43 among men and 27 among women in second and third decades of [13]. Mean number of melanocytic nevi was 13.2 in melasma cases compared to 2.8 in control group in our study. The mean in community can be something in between 2.8 percent and 13.2 percent which is different from other studies. The lower mean number of nevi in control group who lack melasma, seems to be mainly due to coincidence or association between melasma and melanocytic nevi, but the general difference of mean number of nevi compared to other studies can be due to other factors like different sun exposure level, different age distribution among study populations and different genetic or hormonal status among studies.

We found that having freckles, lentigines and having more than three melanocytic nevi were positively related to developing melasma in women increasing the chance of melasma up to 23 times in case of melanocytic nevi.

In spite of our vast literature review, no other study was found to focus on coincidence, association or predictive role of different types of nevi for melasma and only one study was found to report association between nevi and melasma. In a cross-sectional study carried out on 400 pregnant women in Tehran, a correlation was found between number of freckles and nevi on face with melasma [9]. Although the study design was not a suitable one to draw the conclusions as the authors have done, but at least their findings are generally in line with ours.

Freckles are shown to have an autosomal transmission and coincidence with melanocytic nevi [4,14]. It is shown also that there is no genetic influence in simple lentigo. Our main objective of study was to check for possible association between nevi (mainly melanocytic nevi) and melasma to be used for prediction purposes and not to declare a causal relation and we used a method of regression analysis. As we know the concept of regression doesn't generally imply any causal relation between regressor and regressand [15]. Further research may be needed to clarify the validity and specificities of observed association. However if findings of this study are confirmed by large scale prospective studies, existence of some types of nevi specially the lentigines and melanocytic nevi can serve as predictors of melasma. This can be of help in selection of target groups for educational programs regarding melasma prevention.

### Limitations, possible biases and strengths of the study

As like other case-control studies, selection bias is an inevitable part of study. We tried to decrease it by selecting controls on a basis that similar source population is assumed. An acceptable temporality detection is existing in this study due to natural process of nevi and melasma development. That is to say melsma develops later in life than nevi. Although this should be considered as limitation of this retrospective study, but findings of this research encourages researchers to design and conduct cohort studies that can give more reliable findings than case-control studies. Although a precise and much reliable prediction can not be expected for such a case-control study but the strong association and confidence interval function contrary to minimal systematic errors guarantees usefulness of its scientific findings.

## Conclusion

Existence of some types of nevi specially the lentigines and melanocytic nevi increase chance of melasma and may serve as predictors of melasma.

## Competing interests

The authors declare that they have no competing interests.

## Authors' contributions

HA was the leader of research team contributing the A to Z of research and manuscript preparation. HS–B contributed in data analysis and interpretation as well as manuscript preparation. NA contributed in study design, data collection, analysis and interpretation. She also contributed in manuscript preparation. SZ contributed in study design and data collection.

## Pre-publication history

The pre-publication history for this paper can be accessed here:


